# Evaluation of Possible Side Effects in the Treatment of Urinary Incontinence with Magnetic Stimulation

**DOI:** 10.3390/medicina59071286

**Published:** 2023-07-12

**Authors:** Maja Pavčnik, Anja Antić, Adolf Lukanović, Žan Krpan, David Lukanović

**Affiliations:** 1 Faculty of Medicine, University of Ljubljana, 1000 Ljubljana, Slovenia; majapavcnik98@gmail.com; 2 Ljubljana University Medical Center, 1000 Ljubljana, Slovenia; anja.antic35@gmail.com; 3 Division of Gynecology and Obstetrics, Ljubljana University Medical Center, 1000 Ljubljana, Slovenia; adolf.lukanovic@guest.arnes.si; 4 Department of Gynecology and Obstetrics, Faculty of Medicine, University of Ljubljana, 1000 Ljubljana, Slovenia; 5 Independent Researcher, Zanzna s.p., 1000 Ljubljana, Slovenia; zan@zanzna.si

**Keywords:** magnetic stimulation, urinary incontinence, female, treatment, side effects

## Abstract

*Background and Objectives*: Magnetic stimulation is a type of conservative treatment of urinary incontinence. Our aim was to evaluate the possible side effects of this method. *Materials and Methods*: We conducted a systematic literature review. The key search terms were urinary incontinence, magnetic stimulation, and female. All known synonyms were used. Results: 255 titles and abstracts were retrieved, and 28 articles met our inclusion criteria. Out of 28 studies, 15 reported no side effects, five reported side effects, and eight did not report anything. There was no significant difference in the incidence of side effects between the sham and active treatment groups. *Conclusions*: Side effects of magnetic stimulation in comparison to other active treatments are minimal and transient. Among the conservative UI treatment methods, magnetic stimulation is one of the safest methods for the patient and as such a suitable first step in treating UI.

## 1. Introduction

Urinary incontinence (UI) is a common health, hygiene, social, societal, and economic problem, defined since 2002 as any involuntary leakage of urine by the International Continence Society (ICS) [1,2]. The etiology of UI is multifactorial because risk factors include age, pregnancy and childbirth (multiparous women), pelvic floor injury during vaginal delivery, pelvic surgery, menopause (due to decreased estrogen secretion), hysterectomy, increased body weight, lack of physical activity, urinary tract infections, chronic cough, prolonged heavy lifting, congenital weakness of connective tissue, and chronic constipation [1,3,4]. Several types of UI are known, and, based on the basic pathophysiological mechanisms that cause their onset, they are roughly divided into stress UI (urinary incontinence due to pressure or upon exertion), urgency UI (urgency urinary incontinence (UUI)), mixed UI (with characteristics of stress and urge UI), and overflow UI (involuntary release of urine due to an overfull bladder). In practice, however, the borders between different UI types are often blurred due to mixed etiology [1,3,4,5]. Nevertheless, UUI is only part of the syndrome known as overactive bladder (OAB) [1].

Most population studies from various countries have reported that the prevalence of UI ranges from around 25% to 45%. However, this figure is expected to be even higher because many affected women do not even address the problem with their general practitioner or gynecologist [4,6]. Due to the high prevalence of urinary incontinence, a number of treatments have emerged. A detailed diagnostic workup is important to classify the type of urinary incontinence appropriately, which allows for further guidance in the treatment modality. Treatments vary according to the type of urinary incontinence and can range from conservative management to an invasive operative approach [4,7,8]. Recently, laser treatment, Er-YAG and CO2, and magnetic stimulation have been gaining popularity worldwide. The therapeutic role of laser treatment has been at the forefront of research. Despite studies that have confirmed subjective and objective improvements in the symptoms of patients with SUI, there is still a lack of quality evidence in the form of multicentric, randomized, and placebo-controlled studies [9,10,11,12,13,14,15].

On the other hand, magnetic stimulation (MS) is an approach to the conservative treatment of urinary incontinence, which was approved by the FDA in 1998 as a treatment option for UI with pelvic floor muscle stimulation [16]. Given the drawbacks of other conservative treatments for UI, such as the side effects of pharmacotherapy and the invasiveness of electrostimulation, vaginal cones, botulinum toxin A injections, percutaneous stimulation of the posterior tibial nerve (TMS), sacral nerve stimulation, bulking agent injections, and nonabsorbable transvaginal mesh and midurethral slings, research on MS is warranted, considering its inherent advantages: a non-invasive nature and patient acceptability [5].

MS is widely offered as a treatment for UI, although weak evidence of the short-term and long-term effects has been found in systematic reviews (SRs) and meta-analyses. EUA recommendations from 2020 advised not offering magnetic stimulation in the treatment of UI or OAB [17]. However, current EUA recommendations from 2023 no longer contain this statement in the recommendations [18].

## 2. Methods

To evaluate the possible side effects of MS in the treatment of UI, it is first necessary to present the basic principles and effectiveness of magnetic stimulation, magnetic stimulation vs. electrostimulation, and the possible side effects of MS. Because we were interested in determining whether published studies report and prove the side effects of MS in the treatment of UI, we conducted a systematic review.

The international standard Preferred Reporting Items for Systematic Reviews and Meta-Analyses (PRISMA) was used to guide the methodology of this SR [19]. To comprehensively evaluate published studies, we conducted a systematic literature review search using Medline, Pubmed, Embase, Cochrane, and ClinicalTrials. All known synonyms were used for the following key search terms: urinary incontinence, magnetic stimulation, and female. All known synonyms were used for the selected words. We reviewed all research articles, with no lower or upper limit of publication year. The last search was conducted on 28 April, 2023. It should be noted that this article only focuses on research articles. We identified the potentially relevant research articles by examining the abstracts or articles as a whole. Titles and/or abstracts of the studies retrieved using the search strategy were screened independently by two review authors (M.P. and A.A.) to identify studies that potentially met the inclusion criteria of this review. The full text of the potentially eligible studies was retrieved and independently assessed for eligibility by another author (D.L.). Any disagreement over the eligibility of particular studies was resolved through discussion with a fourth author (A.L.). We only focused on studies conducted on female patients, in which an MS stimulator was built into a chair.

## 3. Magnetic Stimulation

### 3.1. Basic Principle of Magnetic Stimulation

**Figure 1:**
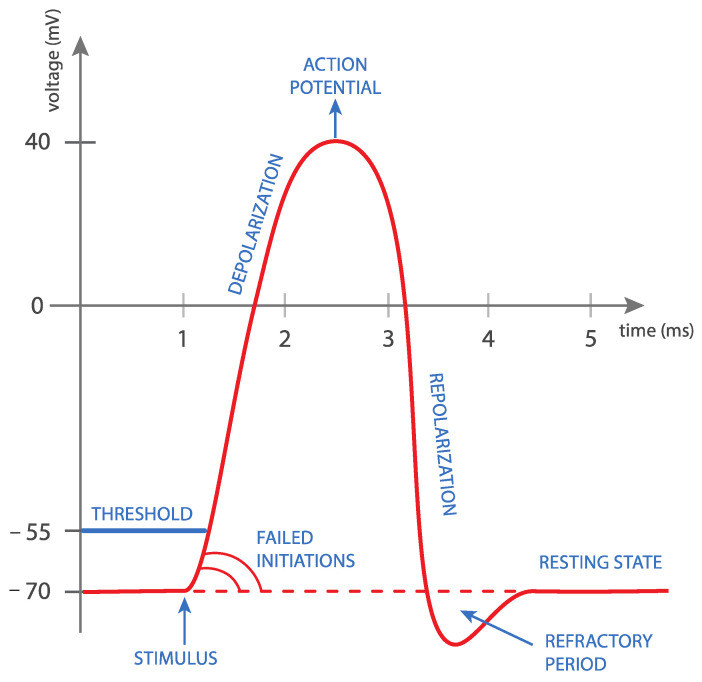
Rapid changes in magnetic field intensity induce an electrical current in the neuron. This phenomenon is called electromagnetic induction. Once the current reaches a certain value, a so-called neuron action potential is achieved. This causes the neuron cell to depolarize, which eventually leads to complete muscle contraction.

**Figure 2:**
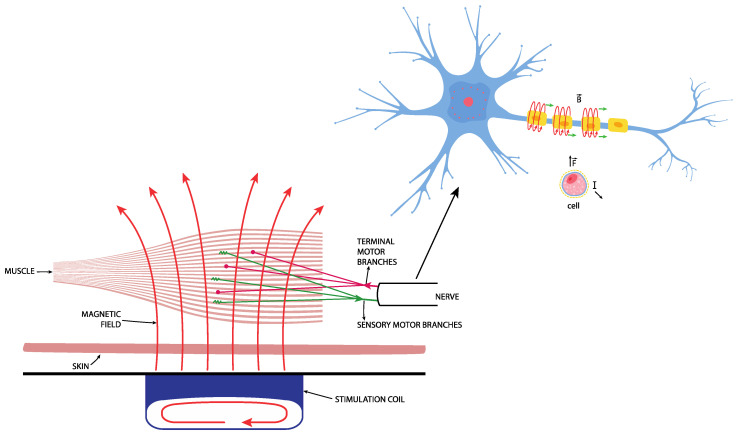
Repetitive effect of magnetic stimulation on the muscle.

MS is the latest method used in the conservative treatment of UI, which is based on Faraday’s law of induction. It follows the principle of magnetic induction, which triggers depolarization of the nerve fibers, which, in turn, causes passive muscle contraction (Figure 1) [16]. The primary goal of this method is to affect the sacral nerves (S2–S4) that innervate the bladder, urethra, vaginal and rectal walls, and pelvic floor muscles [20]. A time modulation of the magnetic current induces an electrical current, which triggers depolarization of the nerve fibers and the consequent contraction of muscle tissue. Repeated activation of the nerve fibers and the subsequent muscle contraction increase muscle strength and endurance (Figure 2). The main and primary target in UI treatment is the afferent fibers of the pudendal nerve, which inhibit the bladder detrusor muscle via the central reflex [16,21]. This inhibition is the result of three activities:

1. Activation of the hypogastric nerve (Lat. *nervus hypogastricus*);
2. Direct inhibition of the pelvic plexus (Lat. *plexus hypogastricus inferior*);
3. Supraspinal inhibition of the detrusor reflex [21,22,23].

A second target important in UI treatment is the efferent nerve fibers, which, when activated, increase pelvic muscle strength and urethral sphincter tone, thus inhibiting the detrusor muscle via the guarding reflex [16]. Ultimately, the repeating muscle contractions work as passive Kegel exercises that stimulate the conversion of fast twitch muscle fibers into slow twitch muscle fibers, making the pelvic floor muscles stronger, more resilient, and more effective (Figure 2) [24].

Because the alternating magnetic field produced affects the afferent and efferent nerve fibers that innervate the bladder, which are located within the produced magnetic field, it can be speculated that, with MS, a better and stronger effect on the entire natural nerve biofeedback loop of the bladder can be achieved.

**Figure 3:**
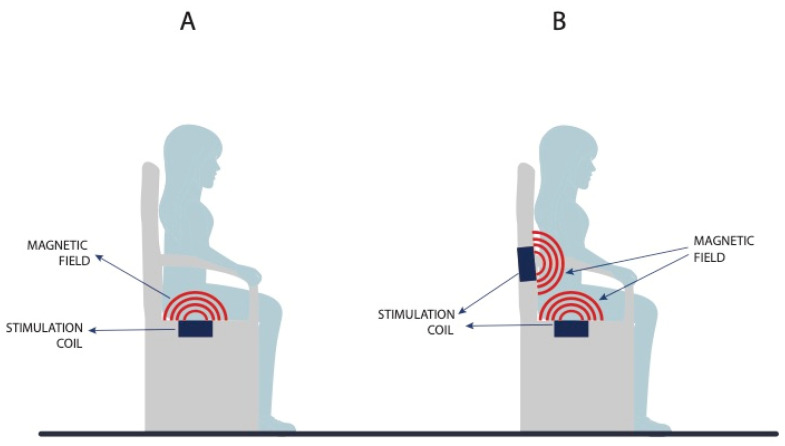
Difference between a magnetic chair with a single magnetic field generator (**A**) and two magnetic field generators (**B**).

The magnetic field penetrates through the fibers without altering them significantly, and the magnetic field magnitude decreases in reverse proportion to the cube of the distance. The magnetic field current also runs uninterruptedly through clothes, and so patients do not have to take their clothes off during therapy. This is one of the advantages of MS compared to other UI treatment methods [25]. At present, UI is treated with MS therapy on a chair, which can contain only one magnetic field generator installed under the seat, and thus the closest to the pelvic floor muscles, or two generators, with the other one installed in the lower part of the backrest, where it is closer to the sacral plexus (S2–S4) (Figure 3). This affects the UUI symptoms and signs [16,24]. Currently, a 3 T magnetic field is in use; this makes it possible to reach the targeted areas such as the sacral plexus and pelvic floor muscles, and, at the same time, as the measurements and information presented in the literature suggest, prevents tissue overload or overheating.

The programs used in medical institutions to treat a specific type of UI differ by both the intensity and frequency of MS. What they have in common is lower and restricted frequencies (up to 35 Hz), which allow for the controlled rehabilitation of damaged tissue and hence prevent deterioration in already damaged tissue. SUI therapy uses higher frequencies because the main problem being addressed is the anatomy of the pelvic floor muscles, their endurance, and their strength. In contrast, the frequencies used in UUI treatment are lower. With UUI, the focus is less on the damage to the pelvic floor muscles itself; rather, the focus is on the functioning of the nervous system and the psychological component, which have a strong impact on the success of MS treatment. The frequencies and intensities during therapies are also lower because we may be dealing with healthy muscles, where only the neural pathway is damaged or the sensitivity of the sensory fibers or the proper regulation of muscle groups is disrupted, which makes it impossible to detect the actual effect of MS on the patient. This can ultimately lead to adverse effects.

The intensity of MS is adjusted individually based on the patient’s sensations, which makes the therapy more patient-friendly and tailored to an individual. There are breaks between individual MS applications to benefit both the device and the patient. The breaks and appropriate cooling methods prevent the device from overheating while retaining the optimal operational conditions during therapy. The breaks also allow for the patient’s muscle tissues to relax, indirectly resulting in appropriate blood circulation in the areas exposed to MS therapy. The flow of fresh blood to the treated tissue supplies the necessary nutrients and removes the waste products generated by muscle contraction during therapy; this increases the effect of MS and reduces the likelihood of side effects.

However, because the programs used to treat UI with MS vary, additional clinical trials are required to standardize them.

### 3.2. Effectiveness of Magnetic Stimulation in the Treatment of UI

The success and effectiveness of treating UI with MS have been both subjectively and objectively proven in many studies. They have mainly been proven subjectively through questionnaires, such as the Incontinence Quality of Life (I-QOL) questionnaire, which showed improved quality of life after treatment with MS, and the ICIQ-SF questionnaire, which demonstrated improvement in symptoms of UI and improved quality of life [23,24]. In addition, the efficacy of treatment has been demonstrated objectively through urodynamic testing, which showed increased bladder volume at first sensation to void, along with increased maximum cystometric capacity and bladder compliance at maximum sensitivity [16,24]. The effectiveness of treatment can also be assessed through a bladder diary analysis, which examines the frequency of urination and the level of urgency before and after therapy. The effectiveness of treating UI with MS is estimated to be between 29–53% and 86–94% for SUI and between 20–25% and 50–85% for UUI [20]. The effectiveness of MS is influenced not only by clinical parameters (the severity and duration of illness, depression, etc.), but also various other factors (age, sex, financial status, etc.), which is why it should be assessed individually, together with the patient’s medical history and clinical picture [26].

A meta-analysis by He et al., which examined 11 randomized controlled trials with a total of 612 patients, showed that MS is a method that decreases UI symptoms, alleviates UI frequency, increases the likelihood of becoming continent, and improves quality of life. It is especially well-suited to patients without sufficient motivation to perform regular pelvic floor muscle training [27]. Yamanishi et al. established that MS is an effective method of treating all types of UI, with recorded symptom improvements in 86% of SUI patients and 75% of UUI patients. Urodynamic testing showed that, in patients with SUI, the maximum intraurethral pressure increased by 34% during stimulation and maximum urethral closure pressure increased by 20.9%. In patients with UUI, significant increases in bladder capacities at first and maximum desire to void during stimulation were noted [20]. Similar findings were also presented by Lo et al., who reported that the efficacy of treatment with MS was 42.1% for SUI and 61.7% for OAB [28]. Lopopolo et al. proved the effectiveness of MS without adverse side effects in patients with MUI. The score of the ICIQ-UI-SF questionnaire decreased by 91% from the baseline, and the score of the ICIQ-OAB and IIQ-7 questionnaires decreased by 86% and 98%, respectively [29]. Gözlersüzer et al. concluded in their SR that MS treatment leads to an improvement in the symptoms of UI, in addition to associated improved quality of life for patients, without any reported side effects [30]. A meta-analysis by Peng et al., which looked at four randomized controlled trials involving a total 232 patients, showed a statistically significant improvement in symptoms in patients with SUI after MS therapy, without any detected side effects. They observed statistically significantly fewer leaks/3 days, less urine loss on a 24 h pad test, higher QoL scores, and lower ICIQ scores [31]. In turn, a systematic review by Antić et al. summarized the efficacy of UUI treatment with MS [5].

Among other things, many clinical trials have also studied long-term improvements after therapy with MS. Yamanishi et al. proved the long-term efficacy of treatment [20]. Similarly, Yokoyama et al. showed that MS cured or improved the condition in 17 out of 20 patients with UUI, and that 53% of patients continued to feel the effect of treatment 24 weeks after the last therapy [32]. Ünsal et al. demonstrated that, after 1 year of treatment, the efficacy of MS is comparable to that of surgery in SUI [33]. In contrast, Doğanay et al. concluded that the effects of treating both SUI and UUI with MS were only temporary, with a high (53%) recurrence rate at 6 months [26]. Bradshaw et al. showed that the effects were only acute and not enduring, whereas Voorham-Vander Zalm et al. described post-treatment changes as statistically insignificant [34,35]. Mikuš et al. recently conducted a randomized controlled trial on the efficacy between Kegel exercises and MS in the treatment of female SUI. They concluded that patients treated with MS had a lower number of incontinence episodes, a better quality of life and higher overall satisfaction with treatment than patients who performed Kegel exercises. No side effects were reported in both groups [36].

After analyzing over 300 articles, the systematic reviews by Lukanović et al. and Antić et al. showed a need for further clinical trials to determine the entry criteria and diagnostic procedures for UI, and to standardize the MS treatment protocols. In addition, longer follow-ups are needed. These are issues that should urgently be addressed in further clinical trials [5,23].

### 3.3. Magnetic Stimulation vs. Electrostimulation

Electrostimulation and MS have been more recent conservative approaches to treating UI. In clinical application, the question often arises regarding which method is more suitable for the patient. Based on the findings in the literature to date, it can be concluded that MS is a good and effective method of treating UI, which causes fewer adverse side effects than electrostimulation [16,22,24,37,38]. Unlike electrostimulation, MS generally does not cause pain. In electrostimulation, the pulse amplitude falls off due to reflections at the boundaries between tissues with different impedance. Thus, to achieve the desired effect, higher electric currents must be delivered to the tissue, thereby activating pain receptors. The density of the magnetic field is the same inside and outside the tissue, which makes it possible to use magnetic fields that do not activate the C pain fibers in the skin [16,22,37]. A comparative study by Yamanishi et al. showed that MS resulted in a greater improvement in symptoms compared to electrostimulation. In addition, overactive detrusor contractions were inhibited in a certain percentage of patients treated with MS; however, the same was not achieved with electrostimulation [38]. The advantages of MS over electrostimulation were also shown by Silantyeva et al., who highlighted the fact that the generated magnetic fields can be used to cause muscle contractions deeper in the tissue [39]. In contrast, Fujishiro et al. established that MS has a stronger effect on the pudendal nerve than ES, which leads to a greater improvement in UI symptoms and signs [40]. A clear advantage of MS over electrostimulation is also evident in the method of application. In MS, the patient sits in the chair without the need to undress, whereas in ES, electrodes are inserted into the vagina or anus, which of course can cause discomfort for the patient. Most importantly, the patient must learn how to use the electrostimulation device properly, whereas with MS she can simply sit in the chair under the operator’s supervision, without the need to learn and know anything in advance. Ultimately, there is also a great difference in terms of side effects: pain, bleeding, urinary tract infections, and mucosal irritation have been observed in electrostimulation, but not MS [20,25,38]. No adverse effects due to continuous MS were noted, which demonstrates its superiority compared to electrical stimulation with regard to pain or noninvasiveness [20].

### 3.4. Possible Side Effects of MS

Possibly the strongest evidence confirming the safe use of MS is the absence of side effects during and after the application of peripheral TMS on patients and after MRI (magnetic resonance imaging), which applies a strong and continuous electromagnetic field through the entire body [25,41]. Even though the research published to date has been dominated by studies that do not record any adverse effects (described below), the interest, in this study, was the possible adverse effects of MS treatment, based on the known facts about the effects of magnetic fields on the human body. Taking into account the theory behind MS, possible side effects could include the following:

1. Muscle overload: During therapies, the muscles that are located in the magnetic field of the MS device are constantly being activated by the device. If the magnetic field or the response of the patient’s muscles is intense, there is a possibility of muscle overload, leading to a longer recovery rate and temporarily weakened muscles.
2. Tissue damage: Although MS is mostly used for tissue repair and faster healing, intense treatment with MS that does not leave adequate time for recovery between pulses/treatments can damage already weakened tissues [42,43,44]. There is a theoretical possibility of electrical overload of the nerve fiber with the induced current. This could only happen if the magnetic field was high enough and directly focused on an already damaged nerve, resulting in an unacceptable level of activity for physiological structures [45]. The actual probability of such a scenario is only theoretical and has never been mentioned or occurred during treatments. It is presumed that such high-voltage exposure would result in visible tissue damage such as burns, and invisible tissue damage such as permanent numbness and/or pain in the skin in the treated area. On the other hand, it is presumed that, if such an electrical overload of the nerve fiber occurred during MS therapy, this resulted in improvements in the final outcome for the patient—presumably by forcing the body to repair the nerve fiber by promoting nerve regeneration with increased blood flow, increasing serum ceruloplasmin expression, improving angiogenesis, and facilitating nerve fiber growth indirectly from vascular tropism. There is also some evidence that this would have positive effects on remyelination [46].
3. Reduction in or loss of sensation on the fibers: If the patient’s nerves and tissue are exposed to an unsuitable duration and/or intensity of magnetic field during MS treatment, the result for the patient could be a tingling sensation, warm skin sensation, poor temperature perception, and so on. The recovery time in such scenarios is brief because it only affects the superficial nerves in the skin.
4. Ineffectiveness of MS therapy: The magnetic field density decreases by the cube root of length, which means that if we increase the distance from the magnetic-field-generating device to a specific point on the patient by a factor of two, we decrease the magnetic field density at the same spot by a factor of eight. We can conclude that the tissue closer to the device generating the magnetic field will always be exposed to a denser magnetic field. Knowledge of the effects and responsiveness of the patient’s body to the magnetic field are crucial when treating deeper areas with MS.
5. Heating or overheating of affected tissues: By exposing the patient’s body to the magnetic field, some energy is transferred to the patient’s body in the form of heat. Very high and intense MS therapies could lead to heating and/or overheating in some parts of the tissue located in the magnetic field. Patients with cardiovascular problems are more affected by this issue because the blood flow is restricted, and thus the tissue-cooling is compromised. More attention should be paid to the patient when there is a risk of seminal fluid being affected by the accumulating heat and when the accumulating heat could lead to an increased risk of vaginal or bladder infection because of the faster development of bacteria in the body. These problems have never been mentioned in the literature and are, therefore, only theoretical. We can conclude that they have a very low probability of occurrence. This kind of side effect is normally avoided by the manufacturers of MS devices with longer pause times in therapy programs that allow for heat to dissipate through the body faster that it can accumulate.
6. The effect of frequencies and magnetic fields on intestinal function and metabolism: During MS therapies, part of the intestine is located in the magnetic field. At present, no accurate measurement can be made determine the overall effect of MS on patients’ intestinal function, organs, and metabolism. The majority of the empirical evidence shows that MS therapy affects patients’ intestinal function in a beneficial way by increasing the metabolic rate, probably mostly through contractions in the surrounding muscles and tissue. Most patients feel a normal need to defecate or urinate after treatments with MS. However, this could lead to an altered metabolic rate, resulting in diarrhea or constipation.

Taking into account the theoretical background of MS, the absolute contraindications for using MS as a method of treatment include pregnancy, certain neurological conditions, active urinary tract infections, and connective tissue diseases.

Despite the theoretical predictions regarding the possible side effects of MS, the main interest was in their occurrence in clinical practice or, specifically, their frequency and type, and their impact on the individual’s treatment satisfaction. In addition, the study also examined the safety of this conservative treatment method, which also greatly depends on the occurrence of side effects.

## 4. Results of Our Literature Review

**Figure 4:**
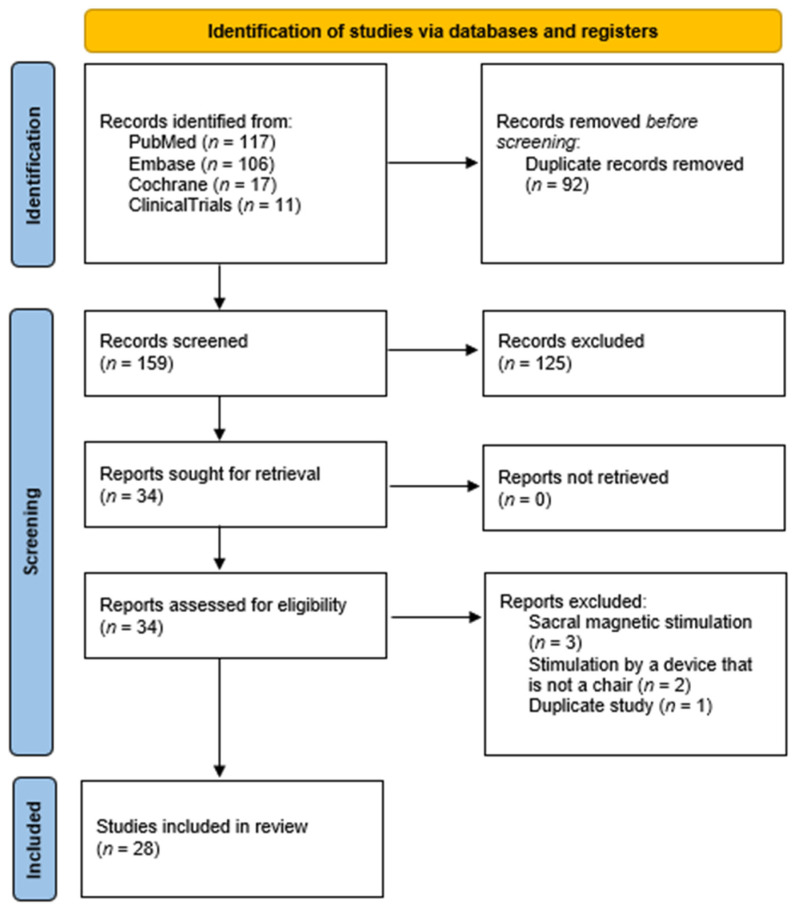
PRISMA flowchart.

A total of 255 titles and abstracts were reviewed, resulting in 141 unique full-text articles in English. In the end, 28 articles that met our inclusion criteria were identified (Figure 4). Fifteen of the included studies, involving a total of 774 patients, reported no side effects, seven of the studies did not mention the monitoring of possible side effects, and one study mentioned monitoring for possible side effects but failed to report whether they were present [16,20,21,23,25,26,28,29,32,33,38,47,48,49,50,51,52,53,54,55,56,57,58]. However, only five studies reported side effects [22,35,59,60,61]. In these five studies, involving 426 patients, side effects were only observed in 56 patients, of whom only 8 were part of the sham group. The results of studies reporting side effects are presented in Table 1.

The prospective, uncontrolled clinical trial focusing on treating SUI with MS conducted by Ismail et al. showed adverse effects in 25 patients (52.1%). Nine patients (18.8%) reported pain in the lower extremities, seven reported abdominal pain, six patients developed cystitis, and six suffered from indigestion. Other reported problems included back and neck pain, palpitations, and paresthesia. The dropout rate was 35.4%. Only in a third of cases was the occurrence of side effects reported as the reason for discontinuing UI treatment with MS [59]. Despite the occurrence of diarrhea and constipation in 16 (15.8%) patients in the active group and three (6.0%) patients in the sham group, Yamanishi et al. concluded that MS is a safe and effective method of treating UI. In addition, they established no statistically significant difference in the occurrence of side effects between the active and sham groups [22].

Lim et al. investigated the efficacy of MS in 120 patients with stress incontinence, establishing side effects in three (5.7%) patients in the active group and five (8.6%) patients in the sham group. They determined no statistically significant differences in the occurrence of side effects between the active and sham groups. The observed side effects included gluteal and pelvic pain, a yellow vaginal discharge, constipation, diarrhea, late periods, and dysuria [60]. A prospective randomized trial by Tezer et al., involving OAB patients with incontinence symptoms, showed no serious side effects. Three (8.5%) patients reported a temporary unpleasant sensation in the pelvic floor, and one patient (3%) reported that she was not feeling well. No patients dropped out of the trial [61].

Voorham et al. reported no side effects expressed by patients, but the EMG measurements of the pelvic floor muscle basal tone showed an increased basal tone in some patients, which they attributed to the effect of MS on the pudendal nerve [35].

The above suggests that the side effects of MS are not serious. It is also interesting that the trials comparing the active and sham groups established no differences in the occurrence of side effects between the two groups [22,60]. Is MS the real reason for this, or does this also involve some other component? It is already widely recognized that the severity of symptoms in SUI depends on previous physical fitness, lifestyle, and the anatomic relations in the pelvis, whereas the psychological component is very important in UUI. The latter can also explain the occurrence of side effects in the sham group. The study by Ismail et al. stands out in this regard because it reported more side effects than any of the other 27 reviewed studies. In addition, only a third of the dropouts were due to side effects [59].

What proves problematic is the fact that eight (28.5%) studies published on this subject did not monitor side effects. Because this therapy has not yet been fully established and included in the guidelines, more high-quality trials and an accurate recording of side effects are needed to objectively assess its safety and efficacy. Because the trials use various therapy programs to treat UI (with various densities and strengths of the magnetic field and various frequencies), it is unknown whether these side effects can be compared. This indicates a need to standardize therapy programs and consistently record side effects. Based on this, therapy programs could be compared and ultimately eliminated; this will provide evidence that MS is a safe and conservative method for treating UI.

Fifteen trials involving a total of 774 patients did not monitor side effects. They concluded that, compared to pharmacological therapy and electrostimulation, MS is a more pleasant method, with fewer side effects, that is non-invasive and safe [16,20,21,26,28,29,32,33,38,49,50,52,53,54,57]. However, the trials that also monitored the side effects showed that these were experienced by 56 patients (in absolute terms). This means that they were reported by only around 13% of all patients involved in trials that also monitored the side effects. Even though the side effects were recorded, they were described as minimal, temporary, and having no effect on the trial’s success rate.

The systematic literature review by Lim et al. showed that, in the trials involving SUI and MS, adverse effects of the therapy were either not present or not monitored, whereas in trials in which UUI was treated with MS, the most frequently reported adverse effects included diarrhea, myalgia, and somnolence. Lim et al. concluded that, among conservative UI treatment methods, MS has rare and mild adverse effects [62]. In a later article, Lim et al. also reported that, despite the occurrence of adverse side effects, patients did not drop out of the trials because they did not experience them as a major burden. This suggests that these side effects are minimal and that MS is tolerable, nonpainful and causes no anxiety in the majority of patients [63].

Due to the vast variability in treatment programs between studies, it is difficult to comprehensively compare and analyze the data. Therefore, no meta-analysis was performed because the studies were clinically diverse, a meta-analysis may lead to biased results and genuine differences in effects may be obscured. Furthermore, many of the included studies lacked a control group, which can limit the validity of the meta-analysis. We only focused on the treatment efficacy and side effects of magnetic stimulation, and we compared it to electrostimulation, which is the most similar treatment modality. To thoroughly assess the pros and cons of each type of conservative treatment, larger reviews should be carried out. Another limitation of our SR could be that only articles published in English were included.

## 5. Conclusions

The increasing awareness among patients that there are other, more conservative methods that can be used, in addition to surgical treatment, facilitates a more preventive approach. In this way, patients will not wait to seek medical assistance when they are already experiencing severe UI symptoms but will opt for preventive approaches and earlier treatment. MS is an effective, safe, and painless treatment method, which allows for the treatment of UI to begin before the symptoms seriously affect an individual’s quality of life [25]. Nonetheless, further clinical trials are needed to more accurately define the optimal duration of MS therapy, suitable stimulation parameters, and a standardized protocol to ensure optimal efficacy. Standardizing the duration of the MS therapy program will make it possible to compare the results of various future trials. Despite the occurrence of side effects, these were not a reason for dropping out of the treatment. Noncompliance with treatment was primarily the result of long-term treatment, which is time-consuming, and a lack of portability. A technical limitation of MS as a method of treatment is also that it is not targeted but, due to the way in which the magnetic field penetrates the tissue, also affects the surrounding tissue. Nonetheless, this method is safe and effective.

Hence, MS could become the first-choice method for treating UI, especially in patients not responding to medication or those for whom medication causes adverse side effects. It could also be the best choice for patients that are not suitable for surgery, do not know how to or refuse to use electrostimulation electrodes, or fail to perform regular pelvic floor exercises [24]. Even though some studies also mention adverse effects of MS treatment, these are minimal and only temporary compared to other conservative treatment methods [62], do not require acute treatment, and do not put the patient’s life in danger. Among the conservative UI treatment methods, MS is one of the safest methods for the patient and, as such, a suitable first step in treating UI.

## Figures and Tables

**Table 1:** Results of studies reporting side effects. n—number, UI—urinary incontinence, SUI—stress urinary incontinence, UUI—urgency urinary incontinence, MUI—mixed urinary incontinence, MS—magnetic stimulation.

	Patients (n)	Type of UI	Treatment Regimen	Side Effects (n, %)	Examples of Side Effects (n)
Yamanishi et al. [22]	151	UUI	Active vs. sham in 2:1 order. Active: 25 min MS, 10 Hz continuously. Sham: 25 min MS, 1 Hz, alternating 5 s on, 5 s off. Twice a week, 6 weeks.	Active: 16 (15.8%) Sham: 3 (6.0%)	Diarrhea (6), constipation (3), myalgia (3), somnolence (3), flatulence (1), muscular weakness (1), pain in extremity (1), limb discomfort (1), back pain (1), …
Voorham et al. [35]	65	SUI, UUI, MUI	21 min MS SUI: 2 × 10 min at 50 Hz, 1-min break in between UUI: 2 × 10 min at 10 Hz, 1-min break in between MUI: 10 min at 10 Hz, 10 min at 50 Hz, 1-min break in between. Twice a week, 8 weeks.	0 patient reported	EMG registered rest tone of the pelvic floor muscles was higher after treatment.
Ismail et al. [59]	48	SUI	5 s on, 5 s off starting at 5 Hz, gradually increasing until 50 Hz. 2 × 10 min at 50 Hz, 2-min break in between. Twice a week, 8 weeks.	25 (52.1%)	Lower limb pain (9), abdominal pain (7), cystitis (6), bowel symptoms (6), backache (5), chair powerful (3), difficult positioning (2), tingling (2), perineal pain (2), neck pain (1), etc.
Lim et al. [60]	120	SUI	Active vs. sham in 1:1 order. 20 min MS Active: 8 s on, 4 s off at 50 Hz. Sham: 8 s on, 4 s off with tilted magnetic coil. Twice a week, 8 weeks.	Active: 3 (5.3%) Sham: 5 (8.6%)	Pain at gluteal muscles and hipbone, yellow vaginal discharge, constipation, diarrhea, mouth ulcer, delayer menstruation, burning sensation or difficulty in passing urine.
Tezer et al. [61]	76	UUI	Bladder training vs. bladder training + MS in 1:1 order. 20 min MS, 10 Hz continuously Twice a week, 6 weeks.	MS: 4 (11.5%)	Temporary discomfort due to pelvic floor pain (3), malaise (1).

## Data Availability

The study does not report any data.
